# The effect of educational intervention based on BASNEF model for reducing internet addiction among female students: a quasi-experimental study

**DOI:** 10.1186/s13052-019-0761-4

**Published:** 2019-12-19

**Authors:** Batol Gholamian, Hossein Shahnazi, Akbar Hassanzadeh

**Affiliations:** 1 MSc in Health Education, School of Health, Isfahan University of Medical Sciences, Isfahan, Iran; 2 Associate Professor, Department of Health Education and Promotion, School of Health, Isfahan University of Medical Sciences, Isfahan, Iran; 3 Instructor, Department of Epidemiology and Biostatistics, School of Health, Isfahan University of Medical Sciences, Isfahan, Iran

**Keywords:** Internet addiction, Student, Education

## Abstract

**Background:**

Adolescents and students are encountered with a challenge so-called “internet Addiction”. This issue affects both their physical and mental health, as well as their academic, social, and family performance. The aim of the current research is to determine the impact of educational intervention. To achieve this aim, BASNEF is utilized to reduce the excessive use of the internet by students.

**Methods:**

This quasi-experimental study was implemented on 120 high school female students in Shahrekord (west of Iran), which were addicted to the internet. Paticipants was randomly divided into two groups of control and intervention. For data collection before and after the intervention, the standard Yang internet Addiction Questionnaire and BASNEF researcher-developed Questionnaire were used. Educational intervention for mothers was conducted in one session (as the most important subjective norm) and for students in two sessions. This method was based on BASNEF construct. Then, data were analyzed using SPSS-20 and chi-square test, Mann-Whitney test, independent t-test, and paired t-test.

**Results:**

After the education intervention, the mean scores of knowledge, attitude, subjective norms, perceived behavioral control and enabling factors in the intervention group were significantly different from the control group (*p* < 0.001). In Post-test assessment, the intervention group revealed a significant decrease, in terms of using the internet (based on the time). (*p* < 0.001).

**Conclusions:**

The results of this study revealed that BASNEF and its related constructs was a suitable framework to design the educational interventions in order to reduce the extreme use of internet in students. Applying of this model can be a cognitive and intellectual framework that affects students’ internet use behavior.

## Background

The internet is one of the most recent human achievements, which is one of the most accessible media, and the most advanced modern communication technologies in the world. The internet provides access to a wide range of sources of information [1]. In 2000–2010, internet users worldwide grew by 44.5%. Currently, the number of internet users is over 36 million in Iran. Internet addiction refers to excessive and uncontrolled use of the internet, which has many negative consequences for youth and adolescents’ academic and social performance [2].

Internet addiction is a major and critical challenge in the adolescents worldwide [3, 4]. With overuse of the internet, they may experience educational problems, inattention, and neglect of family and friends [5].

As a result, the teenagers and youth need to be educated how to use the internet appropriately. Iranian families are moving towards single children while adolescents are experiencing declining social relationships and interactions. Thus, the internet is becoming the most influential factor in their lives [6].. Education can increase students’ awareness of the dangers of excessive use of the internet. When students become aware of the negative effects and consequences of excessive use of the internet, they are less affected by the attraction of the internet [7].

The BASNEF is one of the models, which is utilized to study, identify, and generate new behaviors in society [8].

This model was developed by John Hubly in 1988, which its purpose was to investigate behavior and to plan for change as well as to determine those factors that affect individuals’ decision making. One of the reasons to use this model in the current research is to utilize the constructs of this model as a suitable framework for designing educational interventions that influence students’ addictive behavior on the internet [9].

One of the constructs of the BASNEF is to change the attitudes of students towards the extreme use of the internet. These constructs can be reinforced by appropriating group discussion, playing role and applying pattern students. Question and answer sessions enable students expressing their ideas and beliefs. Students with the help of these sessions believe that excessive use of the internet has many physical and psychological consequences [10].

Another construct is the enabling factors. Providing adequate recreational and sport facilities, creating work, and entertainment for students and occupation security can prevent internet addiction for youth. In Bangladesh, for instance, youth unemployment is high and there is no occupation security, which has led to widespread internet addiction in their community [11, 12].

Behavioral control is another of the BASNEF constructs that can be effective to reduce internet addiction in students. Some factors such as greater communication and social interactions, increased social acceptance of value, and self-esteem in students can reduce their internet usage. This reduction can be achieved using well-planned weekly schedules and using the internet in a controlled and planned behavior [13].

Another constructs of BASNEF is subjective norms. Some issues such as social support and mental security sense in the family and friends are to prevent an extremist accessing to the internet by students. Especially, mothers, as the main pivot of the family, are the most important subjective norm [14]. A study carried out in Jordan has shown that internet addiction is low in adolescents. The reason is that their mothers control and family supervision over the hours and cost of child internet use [15].

**Fig. 1:**
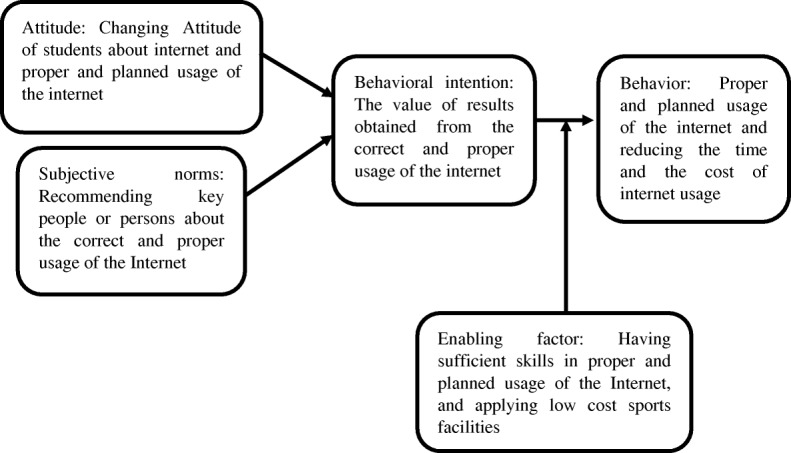
The conceptual framework of this research

Given that the high prevalence of internet addiction among adolescents and students and its negative impact on both their physical and mental health, this research provides a BASNEF-based approach aimed at investigating the effect of educational intervention on reducing internet addiction in Shahrekord girls’ high school students (Fig. 1).

## Methods

### Study design and sampling

This quasi-experimental study was implemented on 120 female high school students of Shahrekord in 2018. Initially, the sample size reached to 88 people with the power factor of 80% and standard error of 5%, using the sample size calculation formula in the intervention studies. However, regarding the possibility of 10% elimination in samples, the number of samples was calculated to be 120. Sampling was carried out in a stage random sampling method, based on which from 10 girls’ high schools in Shahrekord, 6 high schools were selected in the same social and economic situation. There were 712 students in these 6 high schools. Then, the standard questionnaire was distributed among students. After completing the questionnaires and analyzing their results, it was distinguished that 236 students were faced with internet addiction (That is, they used the internet for 8 h or more a day). Afterwards, 120 students were selected by systematic random sampling, and were assigned to control and intervention groups, using random number table. Each mentioned groups contains 60 students.

Parent and student satisfaction, being high school Student, and female gender were considered as inclusion criteria. The exclusion criteria were to be absents in educational sessions and incomplete completion of the questionnaire.

### Data gathering tool

The standard Yang questionnaire was used to determine internet addiction from the students. Knowledge Questionnaire and BASNEF research questionnaire were utilized to investigate the effect of education. In the knowledge section, there were 12 multiple choice questions that gave one score to each correct answer, and zero score to each wrong answer. Therefore, the maximum score was 12 while it was zero at least.

In the BASNEF construct section, which was based on a five-point Likert scale, scored 0 to 4 points. Attitude questions were 4 questions with a maximum score of 16 and a minimum score of zero. For example: “When I feel lonely, I use the internet to talk to others”.

In the Enabling Factors section, there were 4 questions with a maximum score of 16 and at least zero. For example: “Due to lack of facilities, I use the internet to fill leisure time”.

The subjective norms consisted of 6 questions with following choice options: very high, high, low, very low and never. They were rated 0 to 4 based on a Likert scale. Moreover, the maximum score was 24 and the minimum score was zero. For example: “I want to hear the recommendation of my mother about the amount of use of cyber space”.

Perceived behavioral control: it had 4 questions with a maximum score 16 and the score zero, at least. For example: “I can control internet usage hours during the day”.

Behavioral intention: it had 4 questions with a maximum score 16 and the score zero, at least. For example: “I am going to reduce internet usage hours over the next month”.

It should be noted that the questionnaires were completed by the students in both intervention and control groups before and two months after the intervention.

Finally, all questionnaire questions were calculated on the basis of 100 for ease of comparison.

### Validity and reliability evaluation of BASNEF questionnaire

The validity of the questionnaire was assessed by content validity. The initial version of the questionnaire was distributed to 10 health promotion and psychology experts. According their comments, 7 equations were eliminated and 3 questions were added to the questionnaire. At the end, the validity of the questionnaire was calculated and confirmed by Content Validity Index (0.73) and Content Validity Ratio (0.85). Test- retest method was used to evaluate its reliability. In this way, the questionnaire was provided to 15 students with similar characteristics to the target group to complete it.Then, the students completed the questionnaire one week later again. Cronbach’s alpha in different sections (knowledge (0.77), Attitude (0.73), subjective norms (0.80) - perceived behavioral control (0.74) - enabling factors (0.78) were obtained and the reliability of the questionnaire was confirmed.

### Intervention

Before the educational intervention, one session was held for students and their parents about the importance of internet addiction in students and its negative consequences, and the study goals. These issues were described by the researcher. There were no reports of rejection, and all 120 were present by the end of the study. The intervention was according to BASNEF model constructs. A two-day educational session for students and one session for mothers as the most important subjective norm were conducted by a psychologist as follows:

In order to raise knowledge, a lecture about the importance of internet addiction in students and its negative consequences and effects on students’ physical and mental health was presented. Then, they were asked to deliver information on internet addiction in groups of four and to provide further training sessions.

The method of group discussion and question and answer session was used to change attitudes towards internet usage. In this matter, the students posed their own questions and used a group discussion of each other’s experiences with guide a teacher. The aim is to believe that internet addiction has many effects that threaten their physical and mental health. Consequently, it should be able to have a regular and controlled use of the internet.

Regarding the structure of subjective norms that included friends, parents, and especially mothers, who played a central role in the family, educational sessions were held for both mothers and students, based on which the role-playing method was used. To do so, one student played the role of an internet addict who used the internet in an unplanned and excessive manner. One of the mothers, as an influential subjective norm, tried to encourage the student to set up a well-regulated program of internet use. This scenario was prepared by a psychologist.

About the enable factors construct, in the case of importance of sport activities, introducing sports venues with a reasonable cost was suggested. In the case of increasing social interactions with friends and acquaintances, brainstorming method was applied. All students described their views and important comments about the best leisure alternative, which were posted on the whiteboard. It was also expected that the department of education would be required to conclude contracts with appropriate sports facilities and swimming pools at less cost.

Related to the perceived behavioral control construct, students were taught self-regulated behaviors that could be programmed and controlled using the internet. The pattern (role model) students were utilized for this purpose, which had weekly and regular use of the internet and also had low internet use costs. Then, the students were asked to design the best timetable for using the internet by giving the students papers. There was also a short video tutorial on empowering students to adjust their internet usage schedule and to reduce the cost of using the internet.

### Data analysis

SPSS software (Ver20), was used to analyze the results obtained from the questionnaires. Chi-square test was also used to compare the following factors: frequency of parents’ occupations, frequency of internet use between control and intervention groups. Moreover, the Mann-Whitney test was also used to compare the duration of internet use before and after the intervention in both groups. Furthermore, the independent t-test was used to compare the mean scores of knowledge, attitude, subjective norms, enabling factors and perceived behavioral control, before and after the educational intervention, in both groups. Finally, the paired t-test was also used in each group to compare the mean scores of BASNEF construct before and after the intervention.

### Ethical considerations

The project has been approved by the Ethics Committee of Isfahan University of Medical Sciences under code 325739. Initially, the purpose of the study was explained to the participants. Then, the questionnaires were completed, without filling name. Moreover, participants were assured about confidentiality of information.

## Results

The results of Chi-square test indicated that the frequency distribution of mothers’ occupation (*P* = 0.80) and fathers’ occupation (*P* = 0.29) were not significantly different between two groups (Table 1).

**Table 1:** Comparison of demographic characteristics of students in control and intervention groups

Variable		Control Group		Intervention group		*P*-value
		%	No.	%	No.	
Students’ age	16 years old	65	39	58.3	35	0.45
	17 years old	35	21	41.7	25	
Father’s occupation	Employee	23.3	14	30	18	0.29
	worker	11.8	7	11.7	7	
	Business man	58.3	35	50	30	
	Unemployed	3.3	2	0	0	
	Others	3.3	2	8.3	5	
Mother’s occupation	housewife	75	45	78.3	47	0.8
	Employee	15	9	15	9	
	Others	10	6	6.7	4	
Father’s level of education	Elementary	6.7	4	8.3	5	0.89
	guidance	13.3	8	18.3	11	
	High school	41.7	25	31.7	19	
	Academic	38.3	23	41.7	25	
Family income	Low	8.3	5	3.3	2	0.89
	medium	20	12	26.7	16	
	Good	63.3	38	60	36	
	Excellent	8.3	5	10	6	

Mann-Whitney test showed that there was no significant difference between two groups in terms of students’ age (*P* = 0.45), Father’s level of education (*P* = 0.89) and family income (P = 0.89) (Table 1).

Chi-square test revealed that there was no significant difference in frequency of internet usage between two groups (*P* > 0.05) (Table 2).

**Table 2:** Frequency distribution of using internet by students in control and intervention groups

Types of internet Use	Control Group	Intervention group			*P*-value
	%	No.	%	No.	
Searching on internet	41.7	25	35	21	0.45
Scientific research	45	38.3	38.3	23	0.46
Social networks	63.3	58.3	58.3	35	0.57
Spending leisure time	63.3	58.3	58.3	35	0.26
Others	20	13.3	13.3	8	0.33

Mann-Whitney test showed that there was no significant difference between two groups, in the use of internet before intervention (*P* = 0.68). Nevertheless, after the intervention, it was significantly less than the control group, in the intervention group (*P* < 0.001) (Table 3).

**Table 3:** frequency distribution of students’ time usage of internet before and after intervention in control and intervention groups

Time usage	Score	Control Group	Intervention group			*P*-value
		%	No.	%	No.	
	Less than 8 h	0	0	0	0	1
	8 h or more	100	60	100	60	
After Intervention	Less than 8 h	27.7	16	93.3	56	0 < 0.001
	8 h or more	73.3	44	6.7	4	

Independent t-test showed that the mean scores of knowledge, attitude, subjective norms, enabling factors and perceived behavioral control were not significantly different between two groups, before intervention (*P* > 0.05). However, after applying the educational intervention, the mean scores of the mentioned constructs, in the intervention group showed a significant increase as compared to the control group (*P* < 0.001) (Table 4).

**Table 4:** - Comparison of mean scores of knowledge, attitude, subjective norms, enabling factors to reduce internet addiction in control and intervention groups, before and after intervention

		Control Group		Intervention group		*P*-value
		Mean	Standard Deviation	Mean	Standard Deviation	
Knowledge	Before intervention	48.56	23.49	49.16	20.34	0.88
	After intervention	47.36	17.45	74.72	10.56	< 0.001
	*P*-value	0.71		< 0.001		
Attitude	Before intervention	67.74	17.77	69.3	15.19	0.60
	After intervention	66.53	23.04	21.18	9.54	< 0.001
	*P*-value	0.64		< 0.001		
Subjective norms	Before intervention	41.15	20.62	40.08	13.43	0.74
	After intervention	41.98	16.64	64.04	9.36	< 0.001
	*P*-value	0.72		< 0.001		
Enabling factors	Before intervention	28.54	14.44	28.37	14.21	0.95
	After intervention	29.10	15.45	65.27	10.47	< 0.001
	*P*-value	0.79		< 0.001		
Intention to act (about ceasing the internet addiction)	Before intervention	36.76	17.68	35.51	11.39	0.64
	After intervention	37.31	14.88	84.93	9.23	< 0.001
	*P*-value	0.83		< 0.001		

Paired t-test showed that in the intervention group the mean scores of internet knowledge, subjective norms, enabling factors and perceived behavioral control related to internet addiction, after the intervention, were significantly greater than before the intervention. However, after the intervention, the mean score of positive attitude to the internet was significantly lower than before the intervention (*P* < 0.001) (Table 4).

## Discussion

The aim of this study was to determine the effect of educational intervention based on BASNEF constructs on decreasing internet addiction in female students.

The computational results of the study revealed that the mean score of knowledge in the intervention group was significantly increased within two months after the training. This was achieved through the use of planned lectures on the high prevalence of internet addiction among students and their effects on their physical and mental health. This result is consistent with the results of Bogale and Taddeo studies [16, 17].

Students’ attitudes toward the negative consequences of excessive use of the internet and its effects on their physical and mental health were investigated through group discussions among students. After questions were asked, students participated in group discussions. Students changed over two months after the educational intervention regarding excessive and unplanned use of the internet. This suggests that the effectiveness of BASNEF model-based intervention in the area of belief and persistence is to maintain and enhance students’ attitudes toward reducing internet addiction behaviors. These obtained results are consistent with a study conducted by Sarayloo [18].

In this research, the mean score of subjective norms increased within two months after the intervention. This increase was due to the presence of families, especially mothers. Their involvement in the process of changing students’ behavior led to a timely use of the internet. The use of the “role-playing” approach has made students and mothers familiar with the important role of mothers in supporting adolescents and helping them plan for regular and planned usage of the internet. This result is consistent with the results of the work by Perez, Wilson and Askari [19–21].

Regarding the enabling factors mentioned in this study, a significant difference was observed in the intervention group compared to the control group, two months after the educational intervention, indicating a positive role of training in the proper use of leisure time and filling leisure time with the planning. Introducing low-cost gyms, recommending aerobic exercise, coordinating training with the most affordable swimming pools and aerobics can be identified as the most important reasons for changing “enabling factors” in intervention group students. The results of the Bantle and Green study have highlighted the importance of enabling factors to continuous behavior [22, 23].

Perceived behavioral control, in the intervention group had a significant increase compared to before the intervention. It seems that using the role model method in which the template students were used, students were asked to use the internet in a controlled and planned way as well as to use the experiences of the template students. This result is consistent with the results of Shahnazi et al’s study [24].

In this paper, the effect of education on students’ behavioral performance and intention on planned use of the internet was evident. In fact, students’ awareness of the prevalence of internet addiction and its negative consequences increased with the introduction of question and answer sessions and group discussions, and their attitudes toward excessive use of the internet changed. On the other hand, the effect of people on the rational use of the internet increased with the presence of mothers. The above set of interventions using BASNEF constructs led to the creation of a behavioral goal and a change in internet use behavior among students.

The results in India, Kazakhstan, and Burkina Faso are also consistent with the findings of this study [25–28].

## Conclusion

The aim of this study was to reduce internet addiction among students and to conduct educational intervention based on BASNEF. The results confirmed the importance of designing educational intervention using a specific model to reduce the extreme use of internet in students. As the future works, it is suggested to investigate the impact of other educational models on reducing internet addiction in students and adolescents as well as to compare its effectiveness with one of BASNEF.

### Limitations

The current research was conducted on the effect of education on internet addiction reduction using BASNEF model in one of the Iranian cities. So, the results cannot be generalized to all Iranian students.

## Data Availability

Not applicable.
